# Strengthening Global Partnership in Breast Cancer Research

**DOI:** 10.1200/JGO.2016.005264

**Published:** 2016-07-27

**Authors:** Edward L. Trimble, Kathy J. Helzlsouer

**Affiliations:** **Edward L. Trimble** and **Kathy J. Helzlsouer**, National Cancer Institute, National Institutes of Health, Rockville, MD

The accompanying article by Jiagge et al^1^ raises numerous issues critical to global breast cancer research and control. It is important to note that breast cancer is the most common cancer among women as well as the leading cause of cancer deaths among women worldwide. Understanding the different patterns of breast cancer around the globe can facilitate prevention, early diagnosis, and treatment.

The global diaspora of peoples has major implications for health and disease and can provide critical insights for understanding etiology and improving treatment. Historically, for example, women in East Asia have experienced lower risks of breast cancer than women of European descent living in Europe and North America. Studies on women of East Asian descent who migrated to North America, however, have demonstrated rates of breast cancer comparable to those of women of European descent among second-generation immigrants, with intermediate rates among first-generation women. The Michigan-Ghana study team report a higher proportion of triple-negative breast cancer (TNBC) among women in West Africa compared with women of European descent living in North America, with North American women of mixed African and European descent falling in between the two proportions. Because TNBC is most common among women with premenopausal onset of breast cancer, the age distribution of the reported patients with breast cancer is important to consider. The authors state that the rankings persisted among women younger than age 50 years. More recently, the Michigan-Ghana group has expanded their work to include breast cancers among Ethiopian women. Rates of TNBC are much lower among these women compared with those in West Africa, consistent with genetic admixture with peoples of Arab descent. Their preliminary work suggests potential biologic underpinnings, which hopefully will be followed by more expansive investigations of multiple factors. A key remaining question is to what extent these observed differences in the proportion of specific subtypes of breast cancer are a result of underlying genetic factors, constitutional or environmental factors, or, most likely, a combination of these risk factors. Studying differences in exposures across varied populations may help in detecting clues for prevention.

It will be important to link this work with studies of breast cancer among other populations of African descent, both in Africa as well as in the Caribbean and Central and South America. It has been estimated that more Africans were forcibly brought through the slave trade to South America, particularly Brazil, than were brought to North America. We know relatively little about the molecular characteristics of breast cancer in Mozambique and Angola, sites from which many of the Africans taken to Brazil originated. We also need to strengthen research on the molecular characteristics of breast cancer occurring among women of Native American ancestry and those with admixtures of Native American, European, and African descent. Such work will require close collaboration with colleagues across Africa, the Caribbean, and Central and South America.

The work by Jiagge et al^1^ also makes clear the importance of building the infrastructure for diagnosing and treating breast cancer in low- and middle-income countries. One of the first steps is to build awareness among the general public and health care providers, both traditional medicine and allopathic practitioners, of the importance of prompt evaluation of breast masses. Another key step is to facilitate efficient and timely evaluation of breast masses with fine-needle aspiration, core needle biopsies, ultrasound, and diagnostic mammography, accompanied by the appropriate training in biopsy technique, anatomic pathology, and imaging. The presence of a reliable supply chain for biopsy needles, glass slides, and pathology reagents is also critical. The development of the appropriate human resources for health is also central. The multidisciplinary management and complex treatment of breast cancer requires training and career support for lay health workers, nurses, doctors, and technicians. Methods to efficiently deliver complex treatment in an effective and cost-efficient manner are critically needed for low-resource environments. Building the overall capacity of the health care infrastructure in low-resource communities also requires making health care affordable and accessible.

Finally, the article by Jiagge et al^1^ highlights the importance of sustained collaboration and mentorship. The commitment by Dr Newman and her colleagues at the University of Michigan and the Henry Ford Health System has been long-standing. This partnership has helped improve the standard of care as well as broaden our understanding of breast cancer etiology and genetics. We need to encourage many more such models of collaboration to reduce the global burden of breast cancer.
